# Distinct effects of phyllosphere and rhizosphere microbes on invader *Ageratina adenophora* during its early life stages

**DOI:** 10.7554/eLife.95502

**Published:** 2024-06-19

**Authors:** Zhao-Ying Zeng, Jun-Rong Huang, Zi-Qing Liu, Ai-Ling Yang, Yu-Xuan Li, Yong-Lan Wang, Han-Bo Zhang

**Affiliations:** 1 State Key Laboratory for Conservation and Utilization of Bio-Resources in Yunnan, Yunnan University Kunming China; 2 School of Ecology and Environmental Science, Yunnan University Kunming China; Xiamen University China; University of Zurich Switzerland

**Keywords:** plant-microbiome interaction, plant-soil feedbacks, plant-phyllosphere feedbacks, population establishment, plant invasion, seedling mortality, Other

## Abstract

Microbes strongly affect invasive plant growth. However, how phyllosphere and rhizosphere soil microbes distinctively affect seedling mortality and growth of invaders across ontogeny under varying soil nutrient levels remains unclear. In this study, we used the invader *Ageratina adenophora* to evaluate these effects. We found that higher proportions of potential pathogens were detected in core microbial taxa in leaf litter than rhizosphere soil and thus leaf inoculation had more adverse effects on seed germination and seedling survival than soil inoculation. Microbial inoculation at different growth stages altered the microbial community and functions of seedlings, and earlier inoculation had a more adverse effect on seedling survival and growth. The soil nutrient level did not affect microbe-mediated seedling growth and the relative abundance of the microbial community and functions involved in seedling growth. The effects of some microbial genera on seedling survival are distinct from those on growth. Moreover, the *A. adenophora* seedling-killing effects of fungal strains isolated from dead seedlings by non-sterile leaf inoculation exhibited significant phylogenetic signals, by which strains of *Allophoma* and *Alternaria* generally caused high seedling mortality. Our study stresses the essential role of *A. adenophora* litter microbes in population establishment by regulating seedling density and growth.

## Introduction

Plant-modified soil properties affect the performance of plants, which are termed ‘plant-soil feedbacks (PSFs)’. PSFs can affect species coexistence (Bever et al., 1997; van der Putten et al., 2013) and local plant community composition and dynamics (Bauer et al., 2017; Kardol et al., 2007; Teste et al., 2017). For plant invasion, PSFs are usually positive because of escaping soil pathogens and recruiting some beneficial microbes (Liu et al., 2023; Mitchell and Power, 2003; Xu et al., 2012) or negative effects because of accumulating local pathogens (Callaway et al., 2013; Flory and Clay, 2013; Zhang et al., 2020).

Similar to soil, leaf litter can also affect plant growth, species diversity, and community structure (Liu et al., 2017; Ma et al., 2020; Olson and Wallander, 2002), thus playing important roles in population establishment and community dynamics (Jessen et al., 2023; Lamb, 2008; Xiong and Nilsson, 1999). However, related research has focused mainly on physical (e.g. maintaining soil moisture and temperature, increasing nutrition and reducing light) or chemical effects (e.g. releasing allelochemicals) (Demey et al., 2013; Jessen et al., 2023; Möhler et al., 2018; Zhang et al., 2017) but has rarely focused on leaf microbial effects. Until 2017, Whitaker et al., 2017, extended the PSF to aboveground tissues (including leaf, stem, and ﬂoral tissues), termed ‘plant-phyllosphere feedbacks (PPFs)’, and found that all four Asteraceae species experienced stronger negative PPFs than PSFs. Subsequently, this team further verified that all 10 tested Asteraceae plants experienced negative PPFs (Zaret et al., 2021). The lack of strong mutualists and relatively high abundance of pathogens in the phyllosphere may account for the negative PPFs.

In addition to microbial sources (i.e. soil vs leaf litter), ontogeny (seedling growth stage) and soil nutrient levels can affect plant-microbe interactions. For example, seedlings showed distinct sensitivity during the growth stage, and younger seedlings were more susceptible to infection by soil microbes because of fewer defense resources (Geisen et al., 2021; Jevon et al., 2020). Interestingly, leaf litter has an adverse effect on seedling emergence but a positive effect on later plant growth (Abbas et al., 2023; Möhler et al., 2021; Zhang et al., 2022); litter also has a stronger negative effect on earlier vegetation growth than on the elder (Loydi et al., 2013; Wang et al., 2022; Xiong and Nilsson, 1999). Moreover, plants enrich distinct microbes under different nutrient conditions and affect plant performance (Dostál, 2021; Gustafson and Casper, 2004; Widdig et al., 2020). For example, the bacterial diversity in duckweed plants was reduced under nutrient-deficient conditions, but the abundance of Firmicutes increased (Bunyoo et al., 2022), and members of Firmicutes have been reported to promote plant stress tolerance (Xu et al., 2018). Ai et al., 2018, reported that nutrient additions cause crops to enrich some bacteria and fungi from soil and increase yield.

*Ageratina adenophora* (Sprengel) RM King and H Robinson (Asteraceae), known as Crofton weed or Mexican devil weed, has invaded more than 30 countries and areas, including South Africa, Australia, New Zealand, Hawaii, India, and China (Changjun et al., 2021; Julien and Griffiths, 1998). It is a perennial weed and can produce high yields of seeds with a high germination rate (GR) (Lu et al., 2006; Parsons, 1992). This weed commonly grows in monocultures, but a high density of seedlings is not common in the wild. Previous studies have shown that *A. adenophora* can enrich the beneficial soil microbial community to facilitate invasion (Niu et al., 2007; Zhao et al., 2019); in contrast, *A. adenophora* leaves harbor diverse fungal pathogens that can cause adverse effects on itself seed germination and growth (Fang et al., 2019a; Fang et al., 2021a). Thus, it is interesting to determine whether leaf microbes play a distinct role from soil microbes in regulating *A. adenophora* seedling density and whether these effects change with the *A. adenophora* growth stage and soil nutrition level.

In this study, we inoculated *A. adenophora* with soil or leaf litter at three stages, 0 day, 21 days, and 28 days after sowing, and transplanted seedlings to grow in soils with high or low nutritional levels. We first determined the germination, seedling survival, and growth of the *A. adenophora* plants. Then, we characterized the bacterial and fungal communities of the soils and leaf litter as inoculation sources, as well as the microbial communities enriched in the leaves and roots of the *A. adenophora* seedlings after growing; we also isolated the fungi associated with the dead seedlings and tested their seedling-killing effects on *A. adenophora*. Finally, we correlated the microbial community with *A. adenophora* seedling mortality and growth.

We hypothesized that the microbial communities associated with leaf litter and rhizosphere soils can account for the differential effects on *A. adenophora* seedling mortality and growth during different growth stages when growing under different nutrient conditions. We expected that (1) leaf litter would have more adverse effects on seed germination, seedling survival, and growth than soil, as leaf litter often harbors more plant pathogens, and (2) inoculation at different growth stages would change the microbial community enriched by seedlings and thus affect seedling growth. Earlier inoculation had a greater adverse impact on seedling growth than later inoculation, as younger seedlings were more sensitive to pathogen infection than older seedlings. (3) The nutrient level influences seedlings to recruit microbes and thus affects seedling growth.

## Results

### Effects of leaf litter and rhizosphere soil on the mortality and growth of *A. adenophora* seedlings

**Figure 1.**
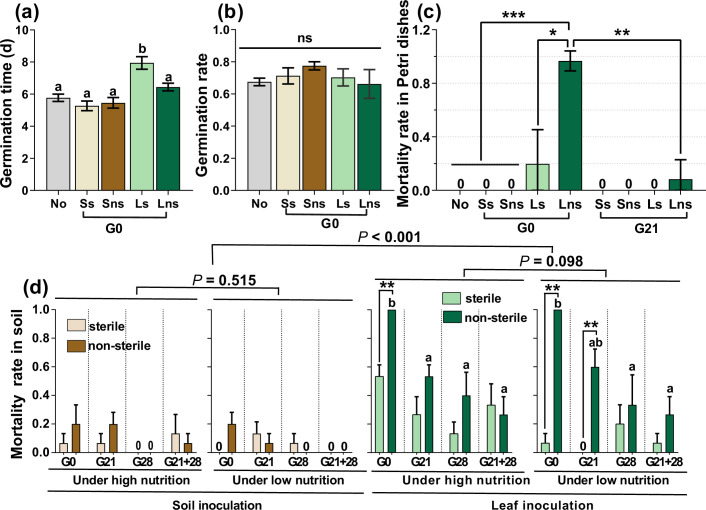
*A.* *adenophora* seed germination and seedling mortality. Seed germination time and rate after soil, leaf or nothing inoculation at the G0 (**a–b**) and seedling mortality rate after soil, leaf or nothing inoculation at the G0 or G21 and subsequent growth in Petri dishes (**c**). Seedling mortality rate after 2 weeks when seedlings were transplanted into soil (**d**). No: nothing inoculated, Ss: sterile soil; Sns: non-sterile soil; Ls: sterile leaf; Lns: non-sterile leaf; G0, inoculated on the day of germination; G21, inoculated on the 21st day after germination; G28, inoculated on the 28th day after germination; G21+28, inoculated on both the 21st and 28th days after germination. *p<0.05, ***p<0.001. Error bars depict the standard error. Different lowercase letters represent significant differences among the different inoculation time treatments (p<0.05). No lowercase letters indicate no differences among the four inoculation time treatments under the same nutrient level (p>0.05).

At the G0 timepoint, sterile leaf inoculation significantly delayed germination time more than did soil and non-sterile leaf inoculation, as well as the control (nothing inoculated) (Figure 1a, p<0.05). Leaf and soil inoculation had no distinct effects on the GR (Figure 1b, p>0.05). In addition, the inoculation of sterile and non-sterile leaves at G0 caused a high mortality rate (MR) (19.7% vs 96.7%) for seedlings growing in Petri dishes. Only non-sterile leaves caused a low percentage of seedling death (8.4%) when the seedlings were inoculated at G21 (Figure 1c, Figure 1—figure supplement 1). Two weeks after transplanting these seedlings into the cups, leaf inoculation caused significantly greater seedling mortality than did soil inoculation (p<0.001); the non-sterile sample caused greater seedling mortality than did the sterile sample, especially leaf inoculation during the G0 and G21 periods. Moreover, non-sterile leaf inoculation at earlier stages significantly increased seedling mortality compared with that at later stages (Figure 1d, p<0.05). However, seedling mortality did not differ between the high and low nutrient conditions, regardless of leaf or soil inoculation (Figure 1d, both p>0.05).

**Figure 1—figure supplement 1.**
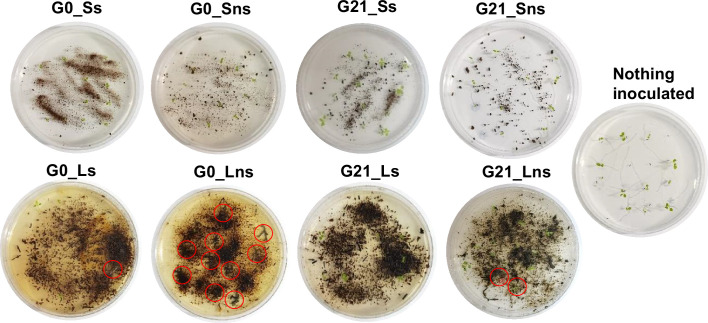
Germination and dead seedlings of *A. adenophora* inoculated soil (upper) or litter (bottom) in Petri dishes. Dead seedlings are circled in red. Ss: sterile soil; Sns: non-sterile soil; Ls: sterile leaf; Lns: non-sterile leaf; G0: inoculated on the day of germination; G21: inoculated on the 21st day after germination. The diameter of the Petri dish is 90 mm.

**Figure 2.**
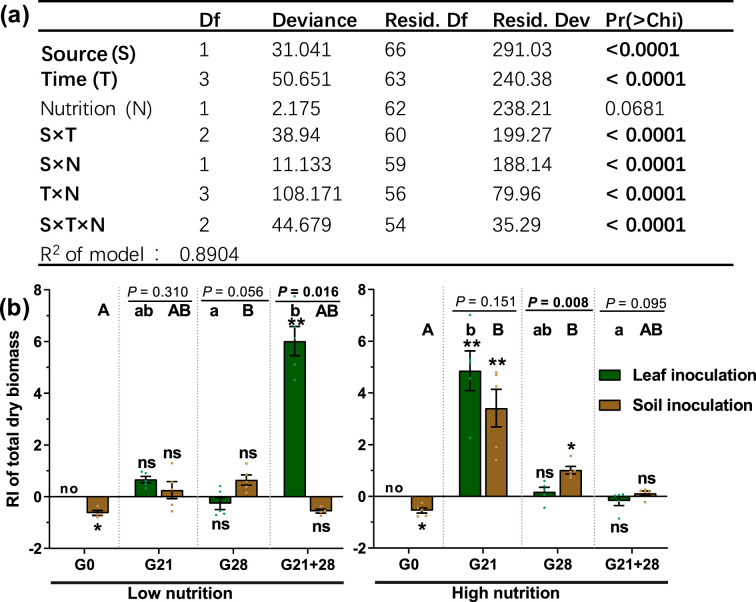
The effects of different treatments and their interactions on seedling growth of *A. adenophora*. Generalized linear model (GLM) analysis (**a**) and comparison (**b**) of the role of microbes in seedling growth based on total dry biomass (response index [RI]) between leaf and soil inoculated at low and high nutrient levels across four inoculation timepoints. Source refers to the leaf or soil inoculation source, time refers to the four inoculation time treatments, and nutrition refers to the nutrient level of the soil. For G0, G21, G28, and G21+28, see Figure 1. Error bars depict the standard error. The difference in the RI among the different inoculation time treatments is indicated by different capital and lowercase letters for the soil and leaf inoculation treatments, respectively. The asterisks indicate that the RIs are significantly different from zero. ‘No’ indicates no surviving seedlings at harvest. p<0.05 is shown in bold.

With the exception of nutrient level, inoculation source and time, as well as their interaction with nutrient level, significantly affected the microbial role in the total dry biomass of *A. adenophora* (p<0.05, Figure 2a). Soil and leaf microbial effects on seedling biomass interacted with soil nutrient level and inoculation time: when inoculated at the G0 timepoint, both soil and leaf microbes had adverse effects on *A. adenophora* growth, as all seedlings inoculated with non-sterile leaves died, and those inoculated with non-sterile soil grew poorer than those inoculated with sterile soil under both low- and high nutrition conditions; when inoculated at the G21 timepoint, both soil and leaf microbes had significantly positive effects only under high nutrition conditions; when inoculated at the G28 timepoint, only soil microbes promoted seedling growth under high nutrition conditions; and when inoculated at the G21+28 timepoint, only leaf microbes had a significantly positive effect on seedling growth under low nutrition conditions (Figure 2b).

### Correlations of the microbial community and potential functions of inocula with *A. adenophora* seedling mortality at the early stage

**Figure 3.**
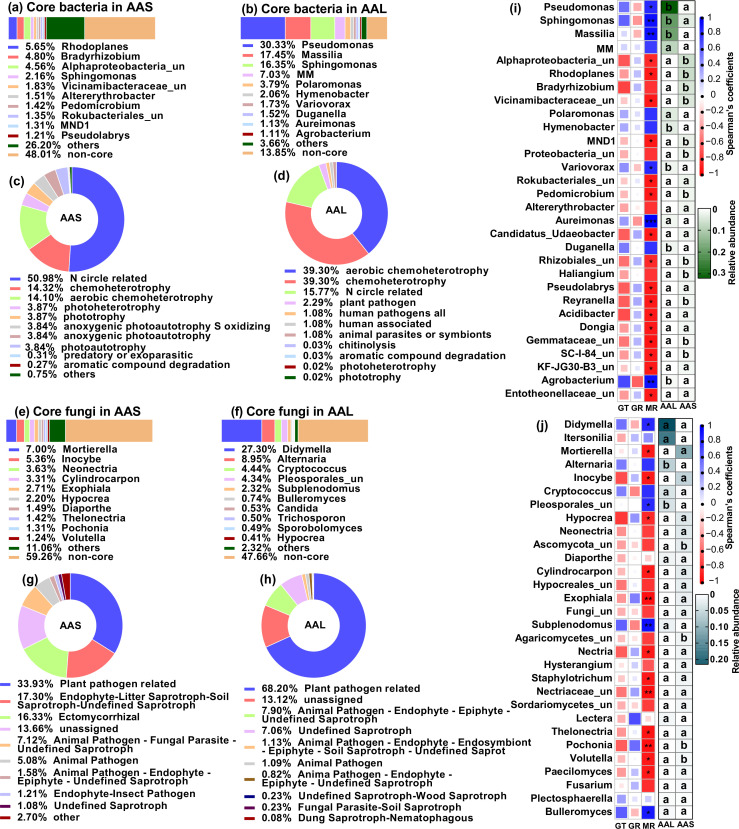
Microbial communities and functions in inoculation sources and their relationship with seed germingation and seedling survival of *A. adenophora*. Microbial community composition and potential functional differences between leaf litter (AAL) and rhizosphere soil (AAS) inocula (**a–h**), as well as correlations of microbial genera with seed germination and seedling mortality (**i–j**). Core bacteria (**a–b**) and fungi (**e–f**) in the AAS and AAL groups. The potential functions of the core bacteria (**c–d**) and fungi (**g–h**) in the AAS and AAL. Correlations of the relative abundance of the top 30 bacterial (**i**) and fungal (**j**) genera in the AAS and AAL with germination time (GT), germination rate (GR), and mortality rate (MR). Only the top 10 core taxa and potential functional groups are shown in the figures. The ‘un’ in the figures is the abbreviation for ‘unclassified’, and the MM is *Methylobacterium-Methylorubrum*. Several bacterial functions classified as N circles-related in the AAL and AAS are shown in Figure 3—source data 1 and 2. Several fungal guilds classified as plant pathogen-related guilds in the AAL and AAS are shown in Figure 3—source data 3 and 4. Red and blue represent negative and positive Spearman’s coefficients, respectively. *p<0.05, **p<0.01, ***p<0.001. Different lowercase letters in the heatmap represent significant differences in the relative abundance of the same genus between AAS and AAL (p<0.05). Figure 3—source data 1.Potential functions of the core bacteria in *A. adenophora* rhizosphere soils. Figure 3—source data 2.Potential functions of the core bacteria in *A. adenophora* leaf litter. Figure 3—source data 3.Potential functional guilds of core fungi in *A. adenophora* rhizosphere soils. Figure 3—source data 4.Potential functional guilds of the core fungi in *A. adenophora* leaf litter.

The soil and leaf inocula had distinct microbial diversity, community compositions, and potential functions. The microbial diversity and richness were greater in the soil than in the leaf litter (Figure 3—figure supplement 1). Top three core bacteria were *Rhodoplanes* (5.65%), *Bradyrizhobium* (4.80%), and some unclassified Alphaproteobacteria (4.56%) for soil and *Pseudomonas* (30.33%), *Massilia* (17.45%), and *Sphingomonas* (16.35%) for leaf (Figure 3a and b). Top three core fungal genera were *Mortierella* (7.00%), *Inocybe* (5.36%), and *Neonectria* (3.63%) for soil and *Didymella* (27.30%), *Alternaria* (8.95%), and *Cryptococcus* (4.44%) for leaf (Figure 3e and f). Plant bacterial pathogens (2.29%) were the potential function of the core bacterial taxa in leaves but not in soil; soil had a greater abundance of nitrogen circle-related function (50.98%) than did leaves (17.55%) (Figure 3c and d, Figure 3—source data 1 and 2). The abundance of plant fungal pathogens was greater in leaves (68.20%) than in soil (33.93%) (Figure 3g and h, Figure 3—source data 3 and 4).

**Figure 3—figure supplement 1.**
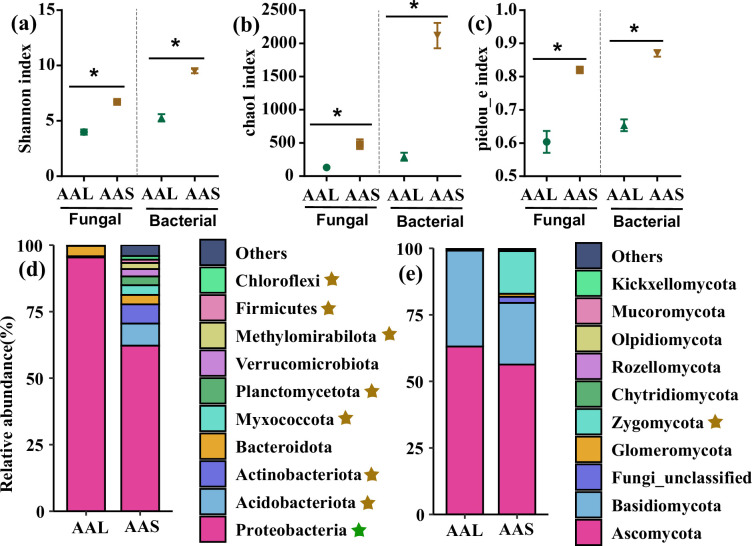
Microbial diversity and community composition at the phylum level in the inoculum AAS and AAL groups. Fungal and bacterial Shannon, Chao 1 and Pielou_e indices of AAS and AAL (**a–c**). Relative abundances of bacterial (**d**) and fungal phyla (**e**) in the AAS and AAL. AAS: *A. adenophora* rhizosphere soil inoculum, AAL: *A. adenophora* litter inoculum. Brown stars represent significantly greater relative abundances in the AAS than in the AAL, green stars represent significantly greater relative abundances in the AAL than in the AAS, p<0.05 at least.

We further correlated the top 30 microbial genera of leaf and soil inocula with seed germination and seedling mortality in response to inoculation with non-sterile inocula at G0. The abundances of both the soil and leaf microbial genera were related to the seedling mortality rate (MR) but not to the germination time (GT) or GR (Figure 3i and j). Specifically, the leaf core bacterial genera *Pseudomonas*, *Sphingomonas*, *Massilia*, *Variovorax*, *Aureimonas*, and *Agrobacterium* were positively correlated with MR, but the soil core bacteria, including Alphaproteobacteria_unclassified, *Rhodoplanes*, Vicinamibacteraceae_unclassified, and *Pedomicrobium*, were negatively correlated with MR (Figure 3a, b, and i). The leaf core fungal genera *Didymella*, Pleosporales_unclassified, *Subplenodomus*, and *Bulleromyces* were positively correlated with MR, but the soil core fungal genera *Mortierella*, *Hypocrea*, *Pochonia,* and *Volutella* were negatively correlated with MR (Figure 3e, f, and j).

**Figure 4.**
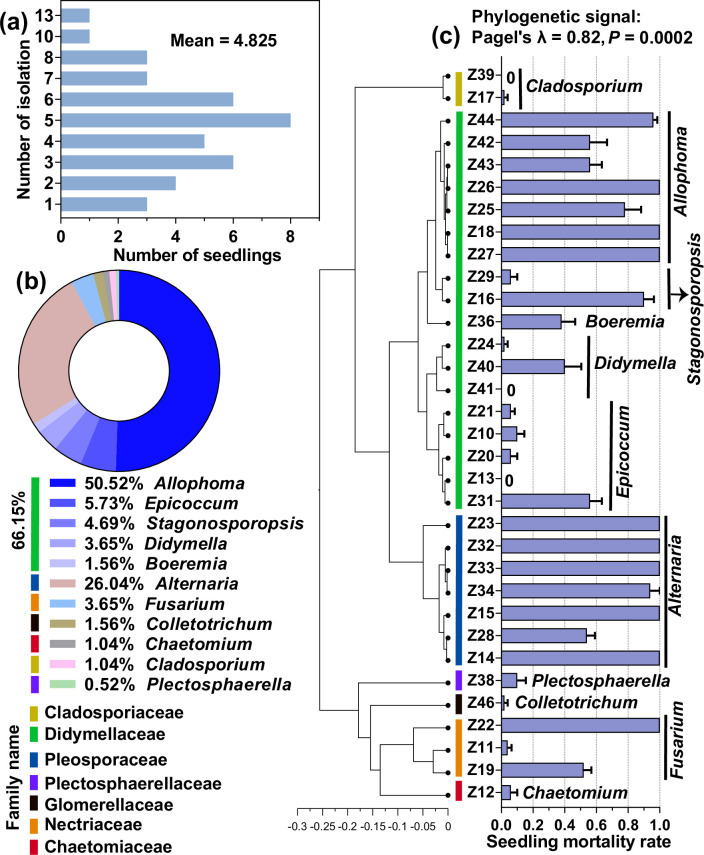
Cultivable fungi associated with dead *A. adenophora* seedlings and their seedling-killing effects on *A. adenophora*. Isolation frequency from one dead seedling (**a**) and cultivable fungal community composition at the genus level (**b**). The seedling-killing effects of 33 fungal strains on *A. adenophora* and their phylogenetic signals (**c**). Figure 4—source data 1.Taxonomy information of 33 representative strains isolated from 40 dead seedlings.

We obtained 192 cultivable fungal isolates from 40 dead seedlings, with an average of 4.825 isolates per dead seedling (Figure 4a). Based on the ITS genes of the representative strains (Figure 4—source data 1), they were divided into seven families. The dominant family was Didymellaceae (relative abundance = 66.15%), and the numerically dominant genera were *Allophoma* (50.52%), *Alternaria* (26.04%), and *Epicoccum* (5.73%) (Figure 4b, Figure 4—source data 1). The seedling-killing effects of these strains on *A. adenophora* exhibited a significant phylogenetic signal (Pagel’s λ=0.82, p=0.0002). Overall, numerically dominant *Allophoma* (Didymellaceae) and *Alternaria* (Pleosporaceae) had high seedling mortality (54–100%) (Figure 4c, Figure 4—figure supplement 1).

**Figure 4—figure supplement 1.**
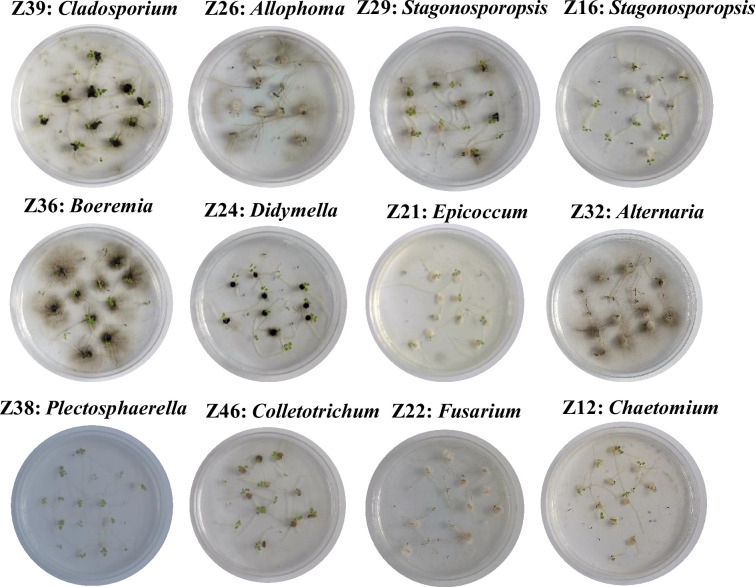
Seedling-killing effects of several representative strains on *A. adenophora*. The diameter of the Petri dish is 9 mm.

### Enrichment of microbial community and function by *A. adenophora* seedlings under different treatments

**Figure 5.**
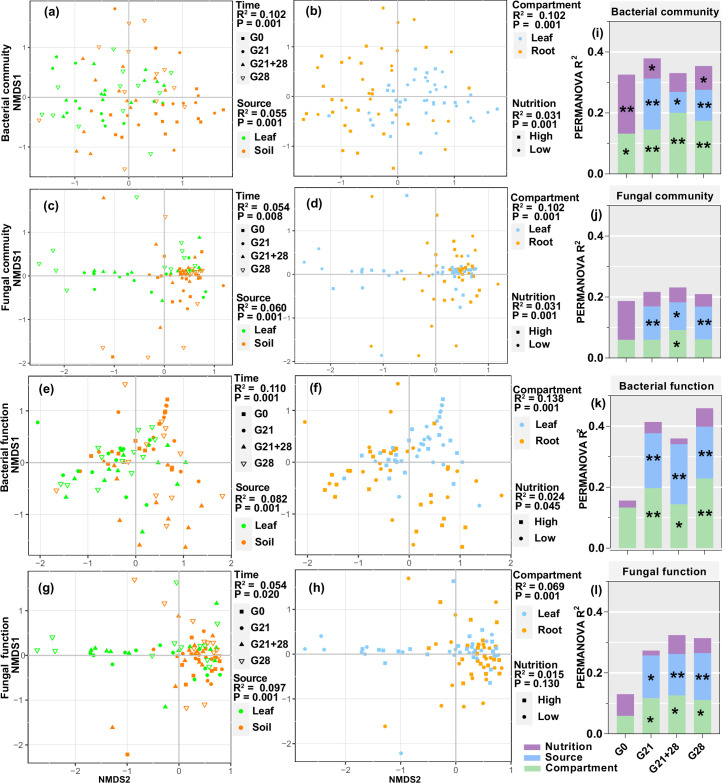
Enrichment of bacterial and fungal communities and functions by *A. adenophora* seedlings under different treatments. Nonmetric multidimensional scaling (NMDS) ordinations of Bray-Curtis dissimilarity matrices with permutational analysis of variance (PERMANOVA) of bacterial and fungal communities and function (**a–h**). Contribution of plant compartment, inoculation source, and nutrient level to the variation in bacterial and fungal communities and function at each inoculation time based on PERMANOVA (**i–l**). *p<0.05, **p<0.01, ***p<0.001. Figure 5—source data 1.PERMANOVA of bacterial communities and function at each inoculation time treatments. Figure 5—source data 2.PERMANOVA of fungal communities and function at each inoculation time treatments.

Nonmetric multidimensional scaling (NMDS) and permutational analysis of variance (PERMANOVA) revealed that all four factors significantly affected the bacterial community and functional assembly of the seedlings, and the greatest effects were inoculation time and compartment (all p<0.05, R^2^: 0.102–0.138), followed by inoculation source and nutrition (all p<0.05, R^2^: 0.024–0.082, Figure 5a, b, e, and f). Additionally, compartment, inoculation source, and time significantly affected the fungal community and functional assembly (all p<0.05, R^2^: 0.054–0.102), but nutrition affected only the fungal community (p=0.001, R^2^=0.031) (Figure 5c, d, g, and h). Further analysis for each inoculation time treatment showed that compartment and inoculation source mainly affected the microbial community and functional assembly and explained a greater proportion of the variation in bacteria than in fungi and in function than in the community. The nutrient level mainly affected the bacterial community at certain inoculation time (p<0.05 for G0, G21, and G28, Figure 5i–l, Figure 5—figure supplement 1, Figure 5—source data 1 and 2).

**Figure 5—figure supplement 1.**
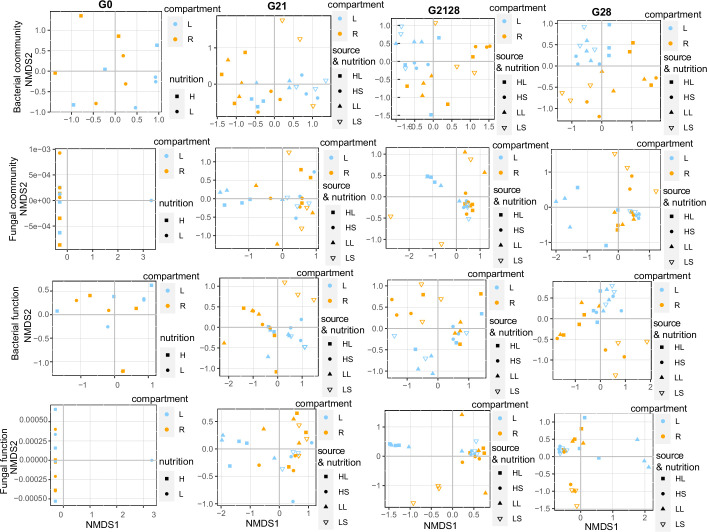
Nonmetric multidimensional scaling (NMDS) results for the bacterial and fungal communities and function in each inoculation time treatment. The different colors represent the different compartments (light blue represents leaves, and orange represents roots). L: leaf; R: root; H: high nutrition level; L: low nutrition level; HL: seedling after leaf inoculation growing under high nutrition level; HS: seedling after soil inoculation growing under high nutrition level; LL: seedling after leaf inoculation growing under low nutrition level; LS: seedling after soil inoculation growing under low nutrition level.

### Correlations of the enriched microbial community and function with *A. adenophora* seedling growth

**Figure 6.**
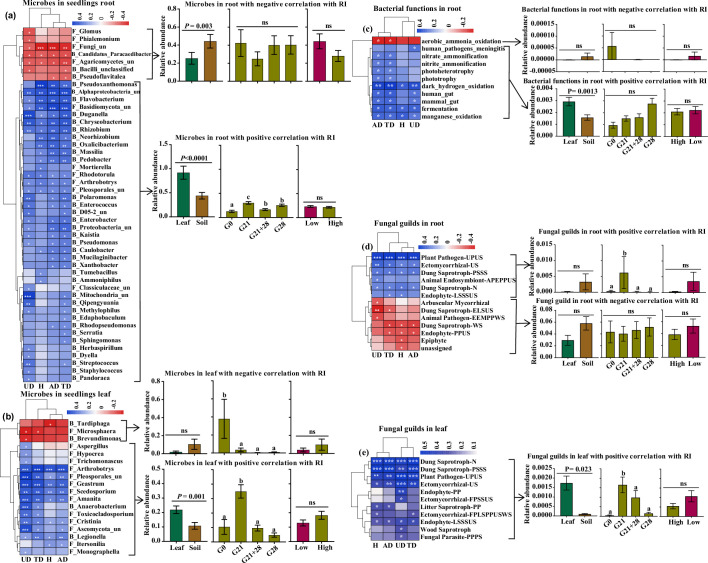
Correlations of the enriched microbial community and function with *A. adenophora* seedling growth. These genera accounted for more than 1% of the total relative abundance in seedling roots or leaves. (**a**) Correlations between 47 out of 214 genera enriched in roots with response index (RI) (left) and the relative abundances of genera under different treatments, i.e., different inoculation sources and time and nutrient levels (right). (**b**) Correlations between 18 out of 184 genera enriched in leaves with RI (left) and the relative abundances of genera under different treatments (right). (**c**) Correlations between putative bacterial functions enriched in roots with RIs (left) and the relative abundances of functions with negative and positive correlations under different treatments (right). (**d**) Correlations between fungal guilds enriched in roots with RIs (left) and the relative abundances of guilds with negative and positive correlations under different treatments. (**e**) Correlations between fungal guilds in leaves with RIs (left) and the relative abundances of guilds with positive correlations under different treatments (right). No bacterial functions in the leaves showed a significant correlation with seedling growth. ‘F_’ represents fungal genera, ‘B_’ represents bacterial genera, ‘un’ represents unclassified; H: RI of seedling height; AD: RI of aboveground dry biomass; UD: RI of underground dry biomass; TD: RI of total dry biomass. Red and blue represent negative and positive Spearman’s coefficients, respectively. *p<0.05, **p<0.01, ***p<0.001. For abbreviations of fungal function guilds, please see Figure 6—source data 1. Figure 6—source data 1.Information about the abbreviations for the fungal functional guilds shown in Figure 6D-E.

We further analyzed the correlation of microbial abundance and putative functions enriched by seedlings with the microbial effect on seedling growth (response index [RI]). We identified 47 root endophytic genera that were significantly correlated with *A. adenophora* growth. Among these genera, seven negative genera were less abundant in seedlings treated by leaf inoculation than in those treated by soil inoculation but were similar in abundance in seedlings treated by different inoculation time, and in seedlings grown at different nutrient levels. In contrast, 40 positive genera, e.g., the fungi *Duganella and Mortierella* and the bacteria *Massilia, Pseudomonas, and Sphingomonas*, were more abundant in seedlings treated by leaf inoculation than by soil inoculation but less abundant in seedlings inoculated at G0 than at the other three inoculation time treatments (Figure 6a). Eighteen leaf endophytic genera with significant correlations with the RI were identified, of which three negative genera, namely, the bacteria *Tardiphaga* and *Brevundimonas* and the fungus *Microsphaera*, were more abundant in seedlings inoculated at G0 than at the other three inoculation time treatments and were slightly more abundant in the seedlings inoculated with soil than in those inoculated with leaf; in contrast, 15 positive genera, e.g., the fungi *Hypocrea* and Pleosporales_unclassified, were more abundant in the seedlings inoculated with leaf than in those inoculated with soil and were more abundant in the seedlings inoculated at G21 than in the seedlings inoculated at the other three inoculation time treatments (Figure 6b).

**Figure 6—figure supplement 1.**
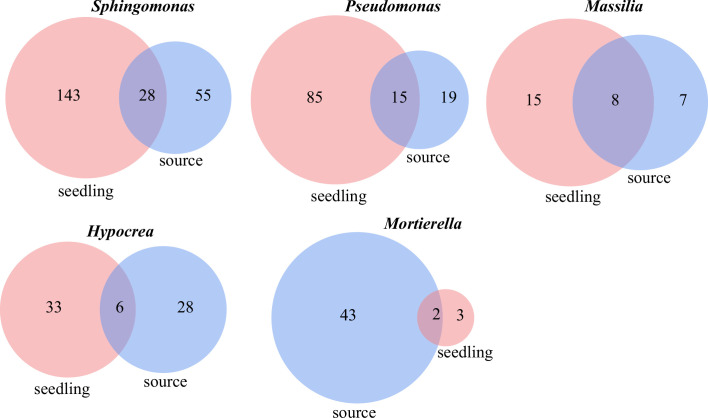
Number of shared amplicon sequence variants (ASVs) between seedlings and inoculum source (soil and litter) of several genera with significant correlation with seedling mortality and growth. Three bacterial genera (upper) and two fungal genera (bottom).

We identified several bacterial functions in the roots and fungal function guilds enriched in the roots and leaves of *A. adenophora* seedlings that were significantly correlated with the RI (Figure 6c–e). Two bacterial functions involved in the N cycle (nitrate ammonification and nitrite ammonification) in roots showed a significant positive correlation with RI (Figure 6c). The fungal guild Ectomycorrhizal showed a significantly positive correlation. Unexpectedly, the putative plant pathogen guild showed a significant positive correlation with seedling growth, while the arbuscular mycorrhizal guild showed a negative correlation (Figure 6d and e). The positive bacterial functions in roots and fungal guilds in leaves had greater relative abundances in the seedlings after leaf inoculation than those after soil inoculation (Figure 6c and e); additionally, the positive fungal guilds in roots and leaves had significantly greater abundances in the G21 seedlings than in those at the other three inoculation time treatments. Surprisingly, there was no difference in the relative abundance of microbes or functions involved in seedling growth under different nutrient levels (Figure 6a–e).

## Discussion

### Leaf litter microbes had more adverse effects on *A. adenophora* seed germination and seedling survival than soil microbes

In support of our first expectation, leaf litter had more adverse effects on seed germination and seedling survival than soil (Figure 1). Leaf litter has been previously reported to have adverse effects on seedling emergence and population establishment (Abbas et al., 2023; Möhler et al., 2021; Zhang et al., 2022) by physical barriers and reducing light and releasing allelochemicals (Abbas et al., 2023; Gross, 1984; Möhler et al., 2018; Zhang et al., 2019). Our study did not directly test the allelopathic effects of leaf litter. However, leaf litter possibly produces allelochemicals that adversely impact *A. adenophora* seed GT and seedling survival. We observed that sterile leaf litter inoculation caused longer GTs than sterile soil and the control (nothing inoculated) (Figure 1a). Interestingly, sterile leaf litter inoculation also caused longer GTs than non-sterile leaf litter inoculation, suggesting that some pathways through which leaf microbes alleviate the adverse effects of leaf allelopathy on GTs are unknown. Moreover, sterile leaf inoculation at G0 caused a 19.7% MR for seedlings growing in Petri dishes (Figure 1c), but no dead seedlings were observed when the plants were not inoculated (Figure 1a, Figure 1—figure supplement 1).

Nonetheless, our study highlighted the adverse microbial role of leaf litter in seedling mortality because non-sterile leaves have significantly greater seedling mortality (96.7%) than sterile leaves (19.7%) when inoculated at G0 (Figure 1c). Indeed, we found that leaf litter harbored more abundant bacterial and fungal genera positively related to seedling mortality, as well as a greater proportion of plant pathogens, than soil (Figure 3). The results implied that the litter harbored many plant pathogens and thus played an essential role in mediating *A. adenophora* population density by killing conspecific seedlings. These findings provide novel insights for understanding plant invasion. Invasive plants are commonly characterized by rapid growth and a high yield of seeds (Baker, 1974; Parsons, 1992; Zheng et al., 2009). These traits are beneficial for rapid population establishment and range expansion at newly invaded sites. However, these invasive plant species commonly form high-density monocultures once the population is established, e.g., *A. adenophora*. For those perennial invasive species, e.g., *A. adenophora*, self-limiting of the population elicited by leaf microbes may alleviate intraspecific competition thus in turn help to maintain monocultures at old established sites. Thus, it is highly interesting to determine whether leaf microbe-mediated self-limitation at an early life stage is common and important in other invasive systems.

### Peripheral microbial sources had more adverse effects on seedling survival and growth when inoculating at the early growth stage than at the later stage

Consistent with our second expectation, inoculation time significantly affected seedling survival and growth; in particular, seedling mortality was greater and seedling growth was poorer when inoculated at G0 than at later growth stages (Figures 1c, d, 2b). The plant growth stage could change the impact of plant host-associated microbes, and such an impact is always strongest in early plant growth stages (Bagchi et al., 2014; Jevon et al., 2020). One potential reason is that small seedlings usually allocate most of their resources to survive and grow, while older seedlings have relatively more resources to defend against pathogen infection (Geisen et al., 2021; Schloter and Matyssek, 2009). For example, smaller seedlings were more sensitive to inoculated individual fungi, soil microbiota, or litter addition than older seedlings due to fewer defense resources and little chance of recovering from biomass loss (Geisen et al., 2021; Jevon et al., 2020; Zhang et al., 2022). Another possible reason is related to the interaction between seed-borne microbes and peripheral microbial sources in young seedlings. There is evidence that seed-borne endophytes are likely to be beneficial for seedling growth and stress resistance (Díaz Herrera et al., 2016; Wang and Zhang, 2023). These seed endophytes might be inhibited or even excluded from young seedlings by external sources of microbes inoculated at G0, when seedlings are highly sensitive to inoculated microbes (Geisen et al., 2021; Jevon et al., 2020). Therefore, determining how peripheral microbial sources interact with seed-borne endophytes in seedlings across ontogeny is highly valuable.

We did not observe an adverse effect of leaf litter microbes on *A. adenophora* growth, as observed previously by Fang et al., 2019b, who inoculated *A. adenophora* at G0 (sowing seeds), because all seedlings inoculated with leaf litter at G0 died after transplantation into soils in this study. In contrast, both the microbial community and function were significantly positively correlated with seedling growth and had a greater relative abundance in seedlings inoculated with leaf litter than in those inoculated with soil, while those with a negative correlation showed the opposite trend (Figure 6). This finding suggested that leaf litter microbes might have a more positive effect on *A. adenophora* growth than soil microbes if inoculated during the latter growth stage, such as at G21 and G21+28 (Figure 2b). Interestingly, we found that N circle-related bacterial functions in seedling roots were positively correlated with seedling growth (Figure 6c). Similarly, Zhao et al., 2019, showed that *A. adenophora* invasion increased N circle-related bacterial functional genes in soil and subsequently directly promoted plant growth and invasion. Fang et al., 2019b, also reported that several root endophytic nitrogen-ﬁxing bacteria of *A. adenophora* could significantly promote its growth. Interestingly, the abundance of these N circle-related bacterial functions was greater in seedling roots inoculated with leaf litter than in those inoculated with soil (Figure 6c), which suggests a possible way in which some N circle-related bacteria associated with leaf litter may migrate from leaves into roots after leaf litter inoculation.

### Nutrient levels did not affect seedling mortality and microbe-mediated *A. adenophora* growth

Nutrient addition can promote severe invasion, as invasive plants often have greater nutrient availability than do native plants (Shan et al., 2024; Slate et al., 2022). However, it is unclear whether such an advantage is involved in a change in the microbial community-driven host growth effect. We also found that *A. adenophora* grew larger and more rapidly in the high nutrient treatment than in the low nutrient treatment (Figure 7—figure supplement 2); moreover, the nutrient level significantly changed the microbial community and bacterial function (Figure 5). Nonetheless, in contrast to our third expectation, seedling mortality was not affected by different nutrient levels, and nutrient levels negligently affected overall microbe-mediated *A. adenophora* growth and the relative abundance of microbes and functions correlated with seedling growth (Figures 1, 2,, 6). Previously, Ai et al., 2018, reported that nutrient additions cause crops to enrich some bacteria and fungi from soil and increase yield; however, there is no evidence that the increased yield effect is due to enriched microbial communities in this study. Our data indicated that the invasion advantage driven by high nutrient availability may be driven primarily by plant physiological traits, such as rapid nutrient absorption and growth strategies, rather than by enriched microbes. Alternatively, it is possible that our delayed harvest of seedlings under low nutrient levels may cover the distinct microbial role in seedling growth between the two nutrient levels (see Materials and methods).

However, there was an interaction effect between nutrient level and inoculation time on seedling growth (Figure 2). For example, a high nutrient level resulted in a more significant positive microbial effect on seedling growth than a low nutrient level when inoculated at G21, regardless of leaf litter or soil inoculation. It is unclear whether, during the first 21 days before inoculation, more beneficial seed endophytes are enriched to produce a more positive effect on seedling growth under high nutrition conditions than under low nutrition conditions, as seed endophytes can facilitate nutrient acquisition and subsequently promote plant growth (Khalaf and Raizada, 2016; Sánchez-López et al., 2018; Shao et al., 2021).

### The same microbial genera had distinct effects on *A. adenophora* seedling survival versus growth

Correlation analysis of the microbial community and function with seedling survival and growth revealed that several genera showed distinct correlations with seedling survival and growth. For example, the bacterial genera *Pseudomonas*, *Sphingomonas,* and *Massilia* are positively correlated with seedling mortality and subsequent seedling growth (Figures 3 and 6). Many strains belonging to these genera have been reported to promote the growth of many plant species (Jiménez et al., 2020; Luo et al., 2019; Qiao et al., 2019), including *A. adenophora* (Chen et al., 2019; Fang et al., 2019a), because they are commonly involved in N_2_ fixation (Albino et al., 2006). However, some microbes, e.g., the fungi *Mortierella* and *Hypocrea*, are negatively correlated with early seedling mortality but positively correlated with later seedling growth (Figures 3 and 6). These fungi, as plant growth-promoting fungi, have been widely reported (Contreras-Cornejo et al., 2009; Ozimek and Hanaka, 2024; Wani et al., 2017). These findings suggested that microbial interactions are highly complicated during the early life stage of *A. adeonophora*. On the one hand, there may be sequential effects for some plant growth-promoting microbial groups. For example, the bacteria *Massilia, Pseudomonas,* and *Sphingomonas* may negatively affect seedling growth and even kill seedlings if the arrival time is too early after germination. On the other hand, such distinct effects of these bacterial groups on seedling survival versus growth may result from different species from the same genus or even from genetically distinct strains from one species. Interestingly, we found that most *Pseudomonas* and *Sphingomonas* amplicon sequence variants (ASVs) enriched in the seedlings (>80%) were not associated with the inoculum source (Figure 6—figure supplement 1). This suggested that most of the bacterial ASVs positively correlated with growth might be from seeds rather than from the inocula. It is necessary to isolate these enriched microbes to test their interactions with the early life stage of *A. adeonophora*.

Surprisingly, related plant pathogen guilds showed a positive correlation with *A. adenophora* seedling growth (Figure 6). Because these putative plant pathogens were classified as plant pathogens based on the current database, it remains to determine whether such putative plant pathogens for most native plant species are not detrimental to invader *A. adenophora* growth. Indeed, plant pathogens often can switch from a beneficial endophyte to a pathogen or vice versa depending on different host plant species (Delaye et al., 2013; Tian et al., 2020).

### Seedling-killing microbes were those associated with leaf litter

We found that most seedling-killing microbes isolated from dead seedlings were previously reported to be leaf spot pathogens. For example, *Alternaria* (Pleosporaceae) and several genera belonging to the family Didymellaceae, such as *Allophoma*, *Stagonosporopsis*, *Didymella*, *Boeremia*, and *Epicoccum*, caused high seedling mortality (Figure 4). *Alternaria* sp. is often pathogenic to a large variety of plants, such as those causing stem cancer, leaf blight, or leaf spot (Leiminger et al., 2015; Thomma, 2003; Mercado Vergnes et al., 2006), and members of *Allophoma* have also been reported to cause dieback (Babaahmadi et al., 2018) and leaf spot (Garibaldi et al., 2012). All these fungi are leaf spot pathogens of *A. adenophora* and its neighboring native plant (Chen et al., 2022; Fang et al., 2021b).

In particular, the numerically dominant *Allophoma* strains obtained in this study had the same ITS genes as the leaf endophyte and leaf spot pathogen *Allophoma* associated with *A. adenophora* (Chen et al., 2022; Fang et al., 2021a; Yang et al., 2023). Interestingly, a previous report revealed that the dominant genera in healthy seedlings inoculated with leaf litter were *Didymella* and *Alternaria* (Fang et al., 2019b). We did not isolate fungi from healthy seedlings to determine whether the live seedlings indeed lacked or accumulated a lower abundance of the seedling-killing strains than did the dead seedlings in this study. We could assume that these fungal genera likely exist in *A. adenophora* mature individual experiencing a lifestyle switch from endophytic to pathogenic and play an essential role in limiting the population density of *A. adenophora* monocultures by killing seedlings only at very early stages. Thus, it is worth exploring the dynamic abundance of these strains and host resistance variation during *A. adenophora* seedling development.

### Implications for developing biocontrol agents for *A. adenophora* invasion

Our data also have implications for the development of biocontrol agents for *A. adenophora* invasion. Currently, several leaf spot fungi, such as the leaf spot fungus *Phaeoramularia* sp., which is released against *A. adenophora* (Kluge, 1991); the white smut fungus *Entyloma ageratinae* against *A. riparia* (Barton et al., 2007); the rust fungus *Uromycladium tepperianum* against the weed *Acacia saligna* (Wood, 2012); and the rust fungus *Puccinia spegazzinii* against *Mikania micrantha* (Day and Riding, 2019), have been used as biological agents for the control of plant invasion. These agents mainly control weeds by damaging the leaves, stems, and petioles and reducing growth rates, flowering, percentage cover, and population density. In this study, the strains associated with leaf litter, such as *Allophoma* sp. and *Alternaria* sp., caused high seedling mortality and thus could control *A. adenophora* invasion at the seedling establishment stage. On the other hand, we found that an external source of microbes had a greater adverse effect on seedling survival and growth when inoculated at G0 than at the later growth stage. Therefore, prevention and control measures by microbial agents taken at the early seedling stage of invasive plants may be more effective than at the mature stage.

## Materials and methods

### Sample collection and preparation

All seeds, rhizosphere soil (AAS) and leaf litter (AAL) of *A. adenophora* were collected from Xishan Forest Park, Kunming city, Yunnan Province (25°55′34″N; 102°38′30″E, 1890 m), on April 9, 2022. We collected dead leaves (litters) from the stems as inoculated leaves to avoid contamination by soil microbes; moreover, dead leaves could better represent litter in natural systems than fresh leaves. All leaf litter and soil samples were collected from five *A. adenophora* populations ∼200 m away from each other and treated as independent biological replicates. These *A. adenophora* plants had been grown in monoculture for more than 10 years; thus, their rhizosphere soils and leaf litters were used in our feedback experiment rather than via a typical two-phase approach (the first conditioning phase and the second testing phase) (Pernilla Brinkman et al., 2010). The collected soil and litter samples were naturally dried in a clean room and weighed. The soil was ground to a 2 mm sieve before weighing. For convenience in the inoculation application, we prepared these samples in leaf litter bags (each containing 2 g of leaf litter) (Zaret et al., 2021) and soil bags (each containing 5 g of soil) as well as 0.1 g of soil or litter (cut into pieces smaller than 2×2 mm^2^) in centrifuge tubes. All sample bags and tubes were divided into non-sterile and sterile groups and stored at 4°C until inoculation. The sterile groups (soils or litter) were sterilized by gamma irradiation (30 kGy, 30 hr, Huayuan Nuclear Radiation Technology Co., Ltd., Kunming, China), which can kill all microorganisms because no colonies were formed after 7 days of inoculation on PDA media for gamma-irradiated samples (see Figure 7—figure supplement 1); moreover, there was no evidence that this irradiation method changed the chemistry of the samples. The non-sterile groups (soils or litter) were natural samples containing live microorganisms. The natural soil or litter (0.3 g) was weighed into tubes and placed at –80°C until DNA extraction.

### Experimental design

In May 2022, all the seeds were surface sterilized, germinated, and subsequently grown in RXZ-380D growth chambers (Ningbo Southeast Instrument Co., Ltd., Ningbo, China) at a temperature of 25/20°C (day/night), a light intensity of 12 000 lux, a 12 hr photoperiod, and a humidity of 65%.

**Figure 7.**
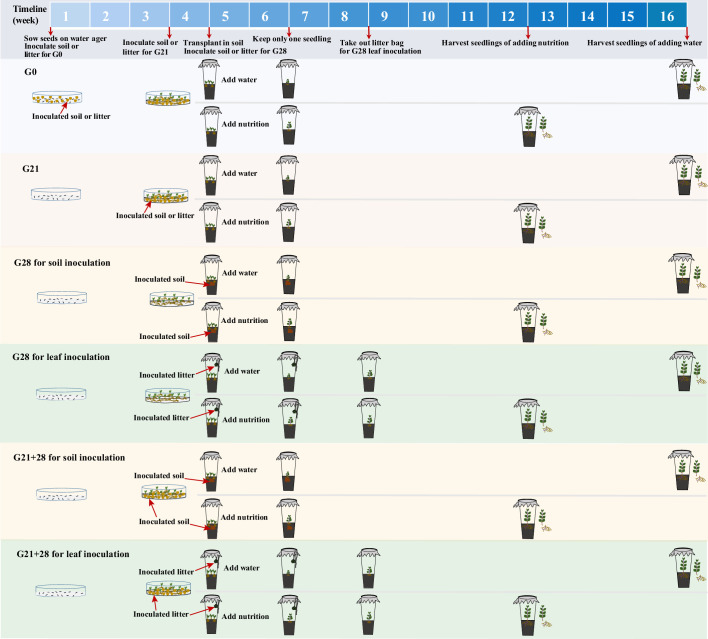
Schematic diagram of soil or litter inoculation at different growth stages. The abbreviations G0, G21, G28, and G21+28 are shown in Figure 1.

The experimental design is shown in Figure 7. (1) We inoculated *A. adenophora* with non-sterile rhizosphere soil and leaf litter of *A. adenophora* and sterile groups as control groups to distinguish the effects of aboveground microbes from those of underground microbes.

**Figure 7—figure supplement 1.**
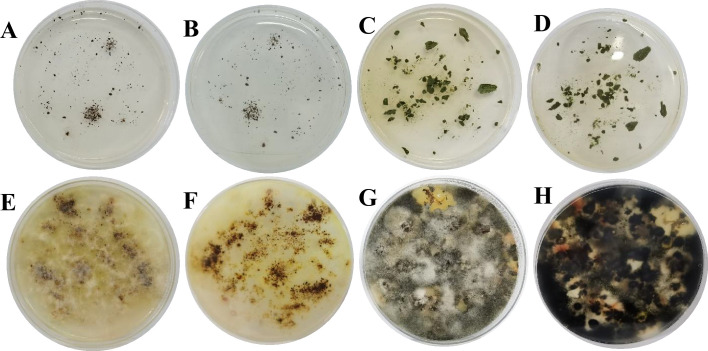
Gamma irradiation was effective at killing all the microorganisms. (A–D) Front and reverse views of gamma-irradiated soil or leaves inoculated on PDA media on the 7th day. (E–H) Front and reverse views of living soil and leaves inoculated on PDA media on the 7th day. The diameter of the Petri dish is 9 mm.

**Figure 7—figure supplement 2.**
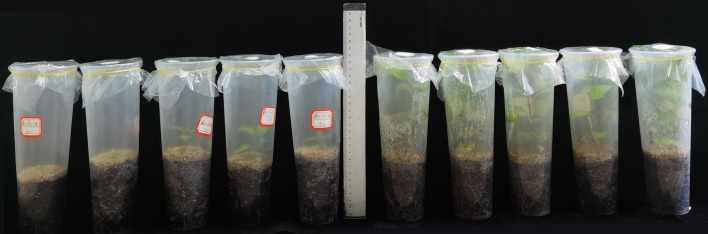
Seedlings were grown for 8 weeks under low (adding water, left) or high (adding Hoagland nutrient solution, right) nutrient levels.

**Figure 7—figure supplement 3.**
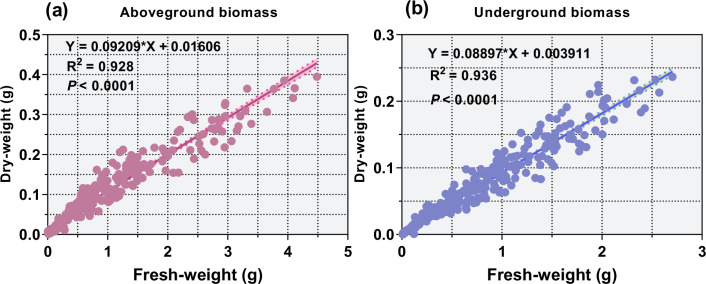
Linear regression between the fresh and dry weights of aboveground biomass (**a**) and underground biomass (**b**).

(2) We inoculated the soils or leaf litters of *A. adenophora* at three growth stages (0 day, 21 days, 28 days) to explore the susceptibility of the seedlings to microbial infection and growth effects. We exposed 16 surface-sterilized seeds to litter or rhizosphere soil from *A. adenophora* when the seeds were sown on a water ager plate (named G0 inoculation). Soils or leaves (0.1 g) were distributed in a thin layer on a plate in 90 mm Petri dishes. Each treatment was replicated five times, resulting in a total of 20 plates (2 inoculum sources × 2 microbial treatments × 5 replicates). We recorded germinated seeds every day from the first to 14th day and on the 21st day after sowing to calculate the GT, GR, and number of dead seedlings on the 28th day. The GT was calculated by the formula GT = Σ(Gi×i)/ΣGi (i: number of days between seed sowing (day 0) and seed germination; Gi: number of seeds germinated on day i), referring to Zhang et al., 2014. The GR was calculated as the proportion of germinated seeds on the 21st day after sowing relative to the total number of sown seeds. The seedling MR=the number of dead seedlings/the total number of germinated seedlings.

Sixteen surface-sterilized seeds were germinated for 21 days in a water agar plate without soil or litter, after which the number of germinated seedlings was recorded. Plates with more than 10 germinated seedlings were chosen for inoculation of 0.1 g of litter or rhizosphere soil (named G21 inoculation), resulting in a total of 20 plates (2 inoculum sources × 2 microbial treatments × 5 replicates). After 7 days of inoculation, germinated and dead seedlings were recorded to calculate the MR.

Sixteen surface-sterilized seeds were germinated for 28 days on a water agar plate without soil or litter, and germinated seeds every day from the first to 14th day and on the 21st day after sowing were recorded to calculate the GT, GR as the control (nothing inoculated). Litter and rhizosphere soil were inoculated when similar-sized seedlings were transplanted into cups after 28 days of germination without soil or litter (named G28 inoculation). For leaf inoculation, 2 g of litter bags was suspended above the plants inside individual cups using twine when three plants were transplanted into the soil in a cup, referring to the study of Zaret et al., 2021, such litter had no direct contact with the soils or plants. Litter (2 g) was placed into individual mesh bags made from 10×15 cm^2^ cheese cloth squares. Litter bags were removed after 4 weeks to avoid the effects of extra allelochemicals. For soil inoculation, 5 g of rhizosphere soil was added to 65 g of sterile background soil when three seedlings were transplanted into the soil in a cup.

Leaf inoculation at G28 was performed to simulate natural microbial spread from the leaf litter to the above part of the seedlings by suspending the leaf bag over the transplanted seedlings without direct contact all the time (see Zaret et al., 2021). This method may result in only microbial species with easy air transmission to infect seedlings. Thus, an additional combination inoculation (named G21+28) was performed to ensure that most leaf microbes had the opportunity to reach the seedlings. Briefly, 0.1 g of litter or rhizosphere soil was inoculated in a plate after 21 days of surface-sterilized seed germination without soil or litter for a total of 20 plates (2 inoculum sources × 2 microbial treatments × 5 replicates). Seedlings were transplanted into the soil in the cup after 7 days of inoculation, and the litter bags were suspended above the plants inside individual cups as part of continuous leaf inoculation. Litter bags were removed after 4 weeks to avoid the effects of extra allelochemicals. Rhizosphere soil (5 g) inoculum was added to 65 g of sterile background soil for soil inoculation.

(3) Seedlings from the four inoculation treatments were respectively transplanted into 1000 mL sterile polypropylene cups containing 65 g of sterile background soil (made of Pindstrup substrate, pearlite, and vermiculite at a volume ratio of 8:1:1, and nutrient content of Pindstrup substrate, see Supplementary file 1) and 120 mL of sterile tap water after 28 days of growth in Petri dishes after sowing. The background soil was sterilized by autoclaving three times for 2 hr, with a 1 day rest period in-between. Three similar-sized seedlings (∼1 cm high with 4 small leaves) were transplanted into each cup and subsequently thinned to one seedling per cup 2 weeks after transplantation to avoid intraspecific competition. We recorded dead seedlings in each cup before thinning. The cups were sealed with PTFE microbial filter membranes to prevent airborne microbe infection and minimize cross-contamination between treatments and randomly placed in the growth chamber and rearranged randomly every week to mitigate potential positional effects. The same volume of water (as low nutrition) or Hoagland nutrient solution (as high nutrition) was added after seedlings were transplanted into cups if needed until seedling harvesting.

In total, our experimental design included 4 inoculation time (G0, G21, G28, G21+28) × 2 inoculum sources (leaf litter, rhizosphere soil) × 2 microbial treatments (sterile, non-sterile) × 2 nutrient levels (high, low) × 5 replicates = 160 cups. Seedlings were harvested after 8 weeks of growth under high nutrient conditions because they grew too fast and touched the PTFE cover; however, we harvested those plants grown under low nutritional conditions after another 4 weeks of growth due to their very small size (see Figure 7—figure supplement 2). No seedlings survived at the G0 inoculation of non-sterile leaf litters when harvested. Stem height, dry aboveground biomass, and underground biomass were measured at harvest. Fresh seedling leaves and roots (0.3 g) from three seedlings per treatment as three replicates were harvested and surface-sterilized and then stored at –80°C until total DNA was extracted for microbial community detection. The aboveground and underground dry biomasses for seedlings with less than 0.3 g fresh weight were obtained by linear regression (see Figure 7—figure supplement 3).

### Molecular sequencing of the microbial community

To link microbial sources (leaf litter and soil) with seed germination, seedling mortality, and subsequent seedling biomass, we sequenced the microbial community associated with inoculum samples (natural AAS and AAL), as well as fresh leaves and roots of *A. adenophora* seedlings grown in the non-sterile treatments.

Total DNA of soil and plant tissue was extracted using the cetyltrimethylammonium bromide (CTAB) method (Stewart and Via, 1993). The quality of the extracted DNA was assessed via electrophoresis in a 1.5% agarose gel using an ND-1000 spectrophotometer (NanoDrop Technology, Wilmington, DE, USA). A Qubit dsDNA HS assay kit (Invitrogen, USA) was used to quantify the DNA concentration. We amplified the bacterial 16S rDNA V4 region and fungal ITS2 region with the primer sets 515F/806R and ITS1F/ITS4, respectively. PCR amplification was performed in a 50 μL mixture containing 12.5 μL of 2× Phanta Max master mix (Thermo Scientific), 2.5 μL of forward primer, 2.5 μL of reverse primer, 50 ng of DNA as a template, and 25 μL of sterile ddH_2_O. The PCR conditions for the bacterial 16S rRNA genes were as follows: 98°C for 30 s, 98°C for 10 s; 35 cycles of 54°C for 30 s, and 72°C for 45 s; and 72°C for 10 min. For the fungal ITS2 region, PCR amplification is performed twice to minimize host plant contamination as much as possible, the first time: 94°C for 5 min, 94°C for 1 min, 20 cycles of 50°C for 50 s, 68°C for 1 min, and 68°C for 10 min; as for the twice: 98°C for 1 min, 98°C for 10 s, 19 cycles of 50°C for 30 s, 72°C for 45 s, and 72°C for 10 min. The PCR products were purified with AMPure XT beads (Beckman Coulter Genomics, Danvers, MA, USA) and quantified with a Qubit (Invitrogen, USA). The amplicon pools were prepared for sequencing, and the size and quantity of the amplicon library were assessed on an Agilent 2100 Bioanalyzer (Agilent, USA) and with the Library Quantification Kit for Illumina (Kapa Biosciences, Woburn, MA, USA), respectively. The libraries were sequenced on a NovaSeq 6000 platform at LC-BIO Biotech Ltd. (Hangzhou, China). High-quality sequences were obtained after removal of low-quality sequences (quality score <20 and sequence length <100 bp). Chimeric sequences were filtered using Vsearch software (v2.3.4). After dereplication using DADA2, we obtained an ASV feature table and feature sequence. The ASV sequences with poor alignment performance and singleton ASVs were discarded. Taxonomic identiﬁcation of bacteria and fungi was performed against the SILVA (v138) (Quast et al., 2013) and UNITE (v8.0) databases (Nilsson et al., 2019), respectively. Alpha diversity was calculated by QIIME2, in which the same number of sequences was extracted randomly by reducing the number of sequences to the minimum of some samples. All the sequences obtained in this study have been deposited in the National Center for Biotechnology Information (NCBI) GenBank under SRA accession number PRJNA1008375 for the bacterial 16S rRNA genes and PRJNA1008403 for the fungal ITS2 genetic region.

### Seedling-killing fungus experiment

No dead seedlings were observed from Petri dishes inoculated with non-sterile soils at G0. Thus, we used 40 dead seedlings obtained from Petri dishes inoculated with non-sterile leaf litter at G0 to isolate fungi. Each dead seedling was cut into 1×1 mm^2^ pieces, and three tissues were placed on each PDA Petri dish and incubated at ambient temperature (20–25°C) for 6–8 days or until mycelia grew. Hyphal tip cultures were subsequently transferred onto new PDA plates and incubated until pure colonies appeared.

Fungal mycelia DNA was also extracted using the CTAB method (Stewart and Via, 1993). We amplified the ITS region of the fungal DNA with the primers ITS4 and ITS5. PCR was performed in a Veriti 96 Well Thermal Cycler (Applied Biosystems Inc, Foster City, CA, USA) in a 50 reactions volume composed of 25 μL of 2× PCR Master Mix, 1 μL of each primer (10 μM), 22 μL of ddH_2_O, and 1 μL of template DNA. The PCR conditions consisted of an initial denaturation at 94°C for 1 min; 35 cycles of denaturation at 94°C for 1 min, annealing at 54°C for 1 min, and extension at 72°C for 1 min; and a final extension at 72°C for 10 min. PCR products were purified, and forward amplicons were sequenced by Sangon Biotech Co., Ltd. (Shanghai, China).

The obtained sequences were edited using EDITSEQ and SEQMAN software in the DNASTAR package (DnaStar Inc, Madison, WI, USA). We aligned sequences in MEGA v6.0 using MUSCLE with default parameters (Edgar, 2004; Tamura et al., 2013), followed by manual checking of alignments. Taxonomic identiﬁcation was performed against the UNITE (v8.0) database, and BLASTN analyses were performed against the GenBank database. The ITS sequences reported in this study have been deposited in the GenBank database (for accession numbers, see Figure 4—source data 1). BEAST v1.10.4 was used to build a Bayesian phylogenetic tree (Drummond et al., 2012). The resulting tree was visualized in FigTree v1.4.3.

To test the seedling-killing effects of these fungal strains on *A. adenophora*, 16 surface-sterilized *A. adenophora* seeds were sown in a water agar plate in a Petri dish. Ten similar-sized seedlings in one Petri dish 21 days after sowing were selected for fungal inoculation. Five Petri dishes were used as five replicates for each strain. Fungi were grown on PDA for 7 days in an incubator at 25°C, after which 3 mm diameter agar discs with fungal mycelia were inoculated into seedlings by touching the leaves or stems (see Figure 4—figure supplement 1). Seedlings were regarded as dead when the leaf and stem became brown and rotten. We recorded the number of dead seedlings after 14 days of inoculation with agar discs and then calculated the MR (=the number of dead seedlings/10).

### Statistical analysis

It is unreasonable to directly compare seedling biomass among treatments because of different harvest time under high or low soil nutrition conditions (see Materials and methods description above), the RI was calculated to evaluate the feedback intensity (or direction) of microbes in the inocula soil or leaf on seedling growth: RI = (variable_non-sterile_ – variable_sterile_)/variable_sterile_ (Bruce Williamson and Richardson, 1988), a one-sample t test was used to determine the significance between the RI value and zero, where RI >0 and <0 represent microbes that promote or inhibit seedling growth, respectively. Because the MRs of some sterile groups were zero and their RIs were impossible to calculate, we had to directly compare the seedling mortality caused by non-sterile with by sterile samples and perform the analysis of correlation between the MR and microbial composition. Generalized linear models (GLMs) with Gaussian error distributions (identity link) generated by the ‘lme4’ package were used to identify the effects of inoculation source, time, nutrient level treatments, and their interaction on the RIs of plant growth. The R^2^ values of the models were obtained by the ‘piecewiseSEM’ package, and p values were estimated using the ANOVA function via chi-squared (χ^2^) tests in GLMs. The nonparametric Mann-Whitney U test was used to perform all two-group comparisons, and the Kruskal-Wallis test was performed to compare the differences in seedling MR, RI, or microbial relative abundances among the four inoculation time treatments.

Nonparametric Mann-Whitney U tests, Kruskal-Wallis tests, and one-sample t tests were performed using SPSS v22.0 (SPSS, Inc, Chicago, IL, USA). NMDS analysis was used to visualize the similarities in bacterial and fungal composition and function among the treatments. PERMANOVA was performed with the ADONIS function in the R (v.4.2.0) package ‘vegan’ to test the differences in the bacterial and fungal communities and functions among the treatment groups. Bacterial functional proﬁles were predicted using functional annotation of prokaryotic taxa (Louca et al., 2016). Fungal functional guilds were inferred using the program FUNGuild, and guild assignments with conﬁdence rankings of ‘Highly probable’ and ‘Probable’ were retained (Nguyen et al., 2016). The core microbial taxa were primarily selected from the ASVs that appeared (100% prevalence) among all the samples. Spearman’s correlation analysis was used to link microbial communities in inoculation sources (leaf litter and soils) with seed germination and seedling mortality in Petri dishes of the non-sterile G0 treatment, as well as to link the RI of seedling growth with microbial communities or functions in seedling leaves or roots by pooling data from all levels of sources, time periods, and nutrients. A correlation was considered significant when the p<0.05. Heatmap plotting was performed in R 4.2.0 with the ‘pheatmap’ package. To examine the phylogenetic signal of the seedling-killing of fungal strains on *A. adenophora*, we calculated Pagel’s λ with the R package ‘phytools’, which measures the distribution of a trait across a phylogeny. A Pagel’s λ closer to 1 indicated a stronger phylogenetic signal (Pagel, 1999). The remaining figures were visualized in GraphPad Prism v7.0 (GraphPad Software, Inc, San Diego, CA, USA).

## Data Availability

The raw bacterial and fungal sequence data are archived in GenBank under accession numbers PRJNA1008375 and PRJNA1008403, respectively. Fungal ITS sequences could be obtained from GenBank under accession numbers OR473386-OR473418. The following datasets were generated: ZhangH 2023foliar endophytic fungi of Ageratina adenophora inoculated by litter and soilNCBI BioProjectPRJNA1008403 ZhangH 2023foliar endophytic bacteria of Ageratina adenophora inoculated by litter and soilNCBI BioProjectPRJNA1008375 ZhangH 2023A complex interaction between microbes and invasive plant Ageratina adenophoraNCBI GenBankOR473418

## Additional files

Supplementary file 1. Fertilizer content in the Pindstrup substrate.

MDAR checklist
