# Supplementary material for: Distinct effects of phyllosphere and rhizosphere microbes on invader Ageratina adenophora during its early life stages
Source: eLife. 2024 Jun 19;13:RP95502. doi: 10.7554/eLife.95502 (PMC11186635; doi:10.7554/eLife.95502)
Supplement: Peer review history

# Editor report: eLife assessment

**DOI:** 10.7554/eLife.95502.3.sa0

**Authors:** Yuxin Chen

**Affiliations:** Xiamen University China

**Keywords:** Solid, Valuable

This study investigates plant-microbe interactions for an invasive plant, Ageratina adenophora. The findings are **valuable** in advancing our understanding of how leaf and soil microbes separately affect its performance, with **solid** experimental evidence revealing the importance of litter microbes in shaping *A. adenophora* populations. The work will be of interest to invasion biologists.

# Referee report: Reviewer #1 (Public Review)

**DOI:** 10.7554/eLife.95502.3.sa1

Summary:

The work by Zeng et al. comprehensively explored the differences in the effects of leaf and soil microbes on the seed germination, seedling survival and seedling growth of an invasive forb, Ageratina Adenophora, and found evidence of stronger adverse effects of leaf microbes on Ageratina compared with soil microbes. By further DNA sequencing and fungal strain cultivation, the authors were able to identify some of the key microbial guilds that may facilitate such negative and positive feedbacks.

Strengths:

(1) The theoretic framework is well-established;

(2) Relating the direction of plant-microbe feedback to certain microbial guild is always hard, but the authors had done a great job in identifying and interpreting such relationships.

Weaknesses:

(1) Allelopathic effects can't be directly accounted for;

(2) The fungal strains accumulated in dead seedlings may also accumulate in live seedlings, thus more evidence is needed to validate the claim by the authors that Allophoma and Alternaria can increase seedling mortality.

# Author comment: Author response

**DOI:** 10.7554/eLife.95502.3.sa2

**Authors:** Zhao-ying Zeng, Jun-Rong Huang, Zi-Qing Liu, Ai-Ling Yang, Yu-Xuan Li, Yong-Lan Wang, Han-Bo Zhang

**Affiliations:** Yunnan University Kunming China; Yunnan University Kunming China; Yunnan University Kunming China; Yunnan University Kunming China; Yunnan University Kunming China; Yunnan University Kunming China; Yunnan University Kunming China

The following is the authors’ response to the original reviews.

**Public Reviews:**

**Reviewer #1 (Public Review):**

Summary:

The work by Zeng *et al*. comprehensively explored the differences in the effects of leaf and soil microbes on the seed germination, seedling survival, and seedling growth of an invasive forb, *Ageratina adenophora*, and found evidence of stronger effects of leaf microbes on *Ageratina* compared with soil microbes, which were negative for seed germination and seedling survival but positive for seedling growth. By further DNA sequencing and fungal strain cultivation, the authors were able to identify some of the key microbial guilds that may facilitate such negative and positive feedback.

Thank you very much for your assessment.

Strengths:

(1) The theoretic framework is well-established.

(2) Relating the direction of plant-microbe feedback to certain microbial guilds is always hard, but the authors have done a great job of identifying and interpreting such relationships.

Thank you very much for your assessment.

Weaknesses:

(1) In the G0 and G21 inoculation experiments, allelopathic effects from leaf litters had not been accounted for, while these two experiments happened to be the ones where negative feedback was detected.

We did not directly test the allelopathic effects. However, we actually also recorded seed germination time (GT) and rate (GR), as well as the seedling mortality rate (MR) for those treatments inoculated soil and leaf after sowing 28 days (G28 inoculation). It is allowed us to observe possible allelopathic effect by comparing sterile sample with control (nothing inoculated during the first 28 days). In this version, we added the result of GT, GR and MR for nothing inoculated (treated as control) in Figure 1, and described results as: “When inoculated at G0 period, the sterile leaf inoculation significantly delayed germination time more than soil and sterile leaves inoculation and control (nothing inoculated) (Fig. 1a, *P* < 0.05)” (see Line102-104). We have also discussed this point in the resubmitted version as: “Our study did not directly test the allelopathic effects of leaf litter. However, leaf litter possibly produces allelochemicals that adversely impact *A. adenophora* seed germination time and seedling survival. We observed that sterile leaf litter inoculation caused longer GTs than sterile soil and the control (nothing inoculated) (Fig. 1a). Interestingly, sterile leaf litter inoculation also caused longer GTs than nonsterile leaf litter inoculation, suggesting that some pathways through which leaf microbes alleviate the adverse effects of leaf allelopathy on GTs are unknown. Moreover, sterile leaf inoculation at G0 caused a 19.7% mortality rate for seedlings growing in petri dishes (Fig. 1c), but no dead seedlings were observed when the plants were not inoculated (Fig. 1a, S1).

Nonetheless, our study highlighted the adverse microbial role of leaf litter in seedling mortality because nonsterile leaves have significantly greater seedling mortality (96.7%) than sterile leaves (19.7%) (Fig. 1c)” in Line 289-301.

(2) The authors did not compare the fungal strains accumulated in dead seedlings to those accumulated in live seedlings to prove that the live seedlings indeed accumulated lower abundances of the strains that were identified to increase seedling mortality.

Thanks for your concerns. We have not isolated fungi from healthy seedlings to make a comparative study. However, our team work previously found that the seedling-killing *Allophoma* strains obtained in this study had the same ITS genes as the leaf endophyte and leaf spot pathogen *Allophoma* associated with mature *A. adenophora* individual; some seedling-killing *Alternaria* also occur in healthy seedlings inoculated by leaf litter. We thus assumed that these seedling-killing fungi, e.g., *Allophoma* and *Alternaria*, likely exist in *A. adenophora* mature individual by a lifestyle switch from endophytic to pathogenic, and these fungi can kill seedling only at very early life stage of *A. adenophora*.

Thus, we discussed this point as: “In particular, the numerically dominant *Allophoma* strains obtained in this study had the same ITS genes as the leaf endophyte and leaf spot pathogen *Allophoma* associated with *A. adenophora* (Chen et al., 2022; Kai Fang et al., 2021; Yang et al., 2023). Interestingly, a previous report revealed that the dominant genera in healthy seedlings inoculated with leaf litter were *Didymella* and *Alternaria* (Kai Fang et al., 2019). We did not isolate fungi from healthy seedlings to determine whether the live seedlings indeed lacked or accumulated a lower abundance of the seedling-killing strains than did the dead seedlings in this study. We could assume that these fungal genera likely exist in *A. adenophora* mature individual experiencing a lifestyle switch from endophytic to pathogenic and play an essential role in limiting the population density of *A. adenophora* monocultures by killing seedlings only at very early stages. Thus, it is worth exploring the dynamic abundance of these strains and host resistance variation during *A. adenophora* seedling development.” in Line 432-

1.

(3) The data of seed germination and seedling mortality could have been analyzed in the same manner as that of seedling growth, which makes the whole result section more coherent. I don't understand why the authors had not calculated the response index (RI) for germination/mortality rate and conducted analyses on the correlation between these RIs with microbial compositions.

Thanks so much. Response index (RI) was calculated as:

(variablenonsterile–variablesterile)/variablesterile. Because mortality rates of some sterile groups were zero values, it is impossible to calculate their RIs. Relatively, only leaf microbes affect seed germination time (GT), leaf and soil microbes did not affect germination rate (GR) (see Fig. 1a,b). Therefore, we preferred to make a direct comparison of the difference between nonsterile and sterile treatments (also see Figure 1d) to assess microbial effect, and we also conducted a correlation by these values with microbial compositions rather than by RIs (see Fig. 3). We emphasized this point in the Materials and Methods in our resubmitted revision as: “Because the mortality rates of some sterile groups were zero and their RIs were impossible to calculate, we had to directly compare the seedling mortality caused by nonsterile with by sterile samples and perform the analysis of correlation between the mortality rate and microbial composition.” in Line 565-568.

(4) The language of the manuscript could be improved to increase clarity.

We have improved language in the resubmitted version.

**Reviewer #2 (Public Review):**

Summary:

The study provides strong evidence that leaf microbes mediate self-limitation at an early life stage. It highlights the importance of leaf microbes in population establishment and community dynamics.

Thank you very much for your assessment.

The authors conducted three experiments to test their hypothesis, elucidating the effects of leaf and soil microbial communities on the seedling growth of *A. adenophora* at different stages, screening potential microbial sources associated with seed germination and seedling performance, and identifying the fungus related to seedling mortality. The conclusions are justified by their results. Overall, the paper is wellstructured, providing clear and comprehensive information.

Thank you very much for your assessment.

**Reviewing Editor (Recommendations For The Authors):**

In addition to the assessments from the reviewers, we have the following comments on your paper:

(1) The experimental design is complicated with regard to the multiple interacting treatments. The statistical analyses show that the interaction terms are important and significant. In this case, it could be more informative to show the detailed results at the sub-level than at the main level in the main text. For example, the main effects of inoculation sources and nutrients shown in Figure 2 are difficult to interpret, because the effects of inoculation sources and nutrients have important dependencies with each other and other factors such as inoculation time as shown in Figure S3. Therefore, Figure S3 is more informative than Figure 2. Please also be cautious that it would be necessary to clarify this context dependence when showing and citing results of the main effect to avoid any possible misunderstanding, such as the case of Figure 2 and S3.

Thanks for your suggestion. We have deleted Figure 2 and placed Figure S3 in the text as Figure 2. And corresponding results have rewritten as “leaf inoculation caused significantly greater seedling mortality than did soil inoculation (*P* < 0.001); the nonsterile sample caused greater seedling mortality than did the sterile sample, especially leaf inoculation during the G0 and G21 periods. Moreover, nonsterile leaf inoculation at earlier stages significantly increased seedling mortality compared with that at later stages (Fig. 1d, *P* < 0.05). However, seedling mortality did not differ between the high- and low-nutrient conditions, regardless of leaf or soil inoculation (Fig. 1d, both *P* > 0.05).” in Line 109-115.

(2) Response index (RI) is already a measure of microbial feedback effect, so that feedback may not be necessary as an explanatory variable in the model with RI as the response variable.

We are sorry that our writing misunderstood you. Here the word “feedback” (e.g., foliage- or soil feedback) does not represent microbial feedback effect, it means leaf or soil inoculation. We have replaced “feedback” by “inoculation source” in the figures and text for better understanding.

(3) Mortality rate is a ratio. It is unclear whether assuming a Gaussian error distribution is fine in your case. It would be important to check the residual distribution and to see whether data transformation (e.g., log) or using other error assumptions (e.g., binomial) is necessary.

Thanks for your suggestion. As you say, it is not appropriate to use generalized linear models (GLMs) with Gaussian error distributions (identity link) to evaluate seedling mortality, because mortality rate is a ratio, which do not meet normality. Thus, we deleted the result of GLM of seedling mortality and directly compared seedling mortality between different microbial treatments, inoculation time, nutrition level and inoculation source by Mann–Whitney U test and Kruskal–Wallis test (see Fig.1 d). All corresponding results have also been rewritten as “leaf inoculation caused significantly greater seedling mortality than did soil inoculation (*P* < 0.001); the nonsterile sample caused greater seedling mortality than did the sterile sample, especially leaf inoculation during the G0 and G21 periods. Moreover, nonsterile leaf inoculation at earlier stages significantly increased seedling mortality compared with that at later stages (Fig. 1d, *P* < 0.05). However, seedling mortality did not differ between the high- and low-nutrient conditions, regardless of leaf or soil inoculation (Fig. 1d, both *P* > 0.05).” in Line 109-115.

(4) Please be consistent about the wording of different treatment names throughout the texts, tables, and figures. For example, "feedback" should only be used for microbial treatment, but not for inoculation source treatment (e.g., Figure 2). We can say there is an effect of microbial feedback only if we compare sterile vs. non-sterile groups, otherwise, there could be other effects, for example, the allelopathic effect pointed out by Reviewer #1. When writing inoculation, please be specific about whether it is for inoculation time or inoculation source (e.g., within multiple statistical tables in the appendix).

Thanks for your good suggestion. We have changed “different feedback” into “different inoculation source” for better understanding our story.

(5) Please clarify which inoculation periods they are for Figures 1d-g.

Thanks for your good suggestion. We have added inoculation periods in Fig.1.

**Reviewer #1 (Recommendations For The Authors):**

Specific comments:

Lines 12-15: This sentence is too long and complicated, making it unclear what had been done and what had not in previous studies.

Thanks a lot. We have reorganized this sentence as: “However, how the phyllosphere and rhizosphere soil microbes distinctively affect seedling mortality and the growth of invasive plants across ontogeny under varying soil nutrient levels remains unclear.”.

Line 19: is it appropriate to use "enrich" here?

Thanks. We have changed “Microbial inoculation at different growth stages altered the microbial community and functions enriched in seedlings” into “Microbial inoculation at different growth stages altered the microbial community and functions of seedlings”.

Line 24-25: "litter exhibited phylogenetic signals"? not clear what this means.

Thanks. Significant phylogenetic signals represent the seedling-killing effects of fungal strains on *A. adenophora* were related to phylogenetic relatedness of these strains. So, we have changed “fungal strains isolated from dead seedlings inoculated with litter exhibited significant phylogenetic signals to seedling mortality” into “the *A. adenophora* seedling-killing effects of fungal strains isolated from dead seedlings by non-sterile leaf inoculation exhibited significant phylogenetic signals, by which strains of *Allophoma* and *Alternaria* generally caused high seedling mortality.”

Line 29: using "in turn" in the first sentence seems weird.

We deleted this.

Lines 32-33: PSFs are usually positive because of?

We have changed “PSFs have positive effects by escaping soil pathogens and recruiting some beneficial microbes” into “PSFs are usually positive because of escaping soil pathogens and recruiting some beneficial microbes”.

Line 54: why emphasize "a single soil microbe"?

Although the research of Geisen *et al*., (2021) assessed the effect of each strain of 34 isolates on seed germination and plant growth, Jevon *et al*., (2020) focused on the soil microbial community on seedling and adult plants survival. Thus, we changed “a single soil microbe” into “soil microbes”.

Lines 85-86: "tested their mortality to seedlings"? not clear what this means.

We are so sorry that our writing misunderstood you. We have changed “we also isolated the fungi associated with the dead seedlings and tested their mortality to seedlings.” into “we also isolated the fungi associated with the dead seedlings and tested their seedling-killing effects on *A. adenophora*.”.

Results: no statistics and no references for the statistical tables that could support the results were presented in this section.

We have deleted the inappropriate generalized linear models (GLMs) with Gaussian error distributions (identity link) for evaluating seedling mortality, and all corresponding results have also described (see Line 109-115 and Fig. 1d).

Lines 100-102: this subtitle reads more like a summary of the following results than a title. All subtitles in the Result section have similar issues (i.e. Lines 148-150, 207-209).

Thanks, we subdivided our Results into four sections and we changed these subtitles as:” Effects of leaf litter and rhizosphere soil on the mortality and growth of *A. adenophora* seedlings”, “Correlations of microbial community composition and potential function with seedling mortality at the early stage”, “Enrichment of microbial community and function by *A. adenophora* seedlings under different treatments”, and “Correlations of the enriched microbial community and function with *A. adenophora* seedling growth”.

Lines 148-206: since there are a lot of results concerning the microbial composition, I suggest focusing on those that could directly explain the positive or negative feedback. The one concerning diversity (e.g. Figure 3 and corresponding texts) does not seem necessary.

Thanks for your suggestion. We have moved figure 3 into the supplementary figures as Figure S2. To focus on core microbes that could directly explain the positive or negative feedback, we reordered Figure 3, where firstly showed the core soil and leaf bacteria, bacterial functions, as well as core soil and leaf fungi, fungal function (Fig3 a-h); and then showed the correlations of top 30 bacterial and fungal genera from soil and leaf with seedling mortality rate (Fig3 i-j).

Line 180: is it not common sense that ectomycorrhiza can only be found in soil?

Yeah, it is. We have deleted this sentence.

Line 199: "the seedling mortality of these strains"? not clear what this means,

We have changed “The seedling mortality of these strains” into “The seedling-killing of these strains on *A. adenophora*”.

Line 291-292: I don't see how the authors can distinguish between allelopathic and pathogenic effects based on their results.

We did not directly test the allelopathic effects. However, we actually also recorded seed germination time (GT) and rate (GR), as well as the seedling mortality rate (MR) for those treatments inoculated soil and leaf after sowing 28 days (G28 inoculation). It is allowed us to observe possible allelopathic effect by comparing sterile sample with control (nothing inoculated during the first 28 days). In this version, we added the result of GT, GR and MR for nothing inoculated (treated as control) in Figure 1, and described results as: “When inoculated at G0 period, the sterile leaf inoculation significantly delayed germination time more than soil and sterile leaves inoculation and control (nothing inoculated) (Fig. 1a, *P* < 0.05)” (see Line102-104). We have also discussed this point in the resubmitted version as: “Our study did not directly test the allelopathic effects of leaf litter. However, leaf litter possibly produces allelochemicals that adversely impact *A. adenophora* seed germination time and seedling survival. We observed that sterile leaf litter inoculation caused longer GTs than sterile soil and the control (nothing inoculated) (Fig. 1a). Interestingly, sterile leaf litter inoculation also caused longer GTs than nonsterile leaf litter inoculation, suggesting that some pathways through which leaf microbes alleviate the adverse effects of leaf allelopathy on GTs are unknown. Moreover, sterile leaf inoculation at G0 caused a 19.7% mortality rate for seedlings growing in petri dishes (Fig. 1c), but no dead seedlings were observed when the plants were not inoculated (Fig. 1a, S1).

Nonetheless, our study highlighted the adverse microbial role of leaf litter in seedling mortality because nonsterile leaves have significantly greater seedling mortality (96.7%) than sterile leaves (19.7%) (Fig. 1c)” in Line 289-301.

Lines 383-414: Correlations are not necessarily causations. Sometimes a strong correlation may result from higher-order interaction. The authors should be more cautious about the discussion of microbial function in this section.

Thanks. We deleted all descriptions of adverse effect or beneficial effect on host plant *A. adenophora* growth and cautiously used “negative correlation or positive correlation” to discuss the functions of these enriched microbes by *A. adenophora*. In the last, we also added a sentence to say: “It is necessary to isolate these enriched microbes to test the interactions with the early life stage of *A. adeonophora*.”

(see Line 411-413).

Lines 489-490: I don't really understand why the authors performed a combination treatment. What did they expect from such a combination?

Thanks. We described our consideration as: “Leaf inoculation at G28 was performed to simulate natural microbial spread from the leaf litter to the above part of the seedlings by suspending the leaf bag over the transplanted seedlings without direct contact all the time (see Zaret et al. (2021)). This method may result in only microbial species with easy air transmission to infect seedlings. Thus, an additional combination inoculation (named G21+28) was performed on both the 21st (with seedling contact) and 28th days (without seedling contact) to ensure that most leaf microbes had the opportunity to reach the seedlings.” see Line 498-505.

Figure 1: why not use "mortality rate" instead of "death rate"?

Thanks. We have changed “death rate” into “mortality rate” in all corresponding figures and text.

Figure 8: This is a very complicated experimental setup. Why did the authors harvest the plants treated with nutrient addition after the 12th day of the experiment and harvest those without nutrient addition after the 16th day? Why the time lag?

Thanks. We explained this as: “Seedlings were harvested after 8 weeks of growth under high-nutrient conditions because they grew too fast and touched the PTFE cover; however, we harvested those plants grown under low-nutritional conditions after another 4 weeks of growth due to their very small size (see Fig. S6).”

(see Method in Line 514-517).
